# Persistent negative symptoms in young people at clinical high risk of psychosis treated with an Italian early intervention program: a longitudinal study

**DOI:** 10.1007/s00406-024-01808-w

**Published:** 2024-04-26

**Authors:** Camilla Ricci, Emanuela Leuci, Emanuela Quattrone, Derna Palmisano, Pietro Pellegrini, Marco Menchetti, Simona Pupo, Lorenzo Pelizza

**Affiliations:** 1 Department of Biomedical and Neuromotor Sciences, “Alma Mater Studiorum” Università degli Studi di Bologna, Via Pepoli 5, 40123 Bologna, Italy; 2 Department of Mental Health and Pathological Addictions, Azienda USL di Parma, Largo Palli 1/a, 43100 Parma, Italy; 3 Division of Pain Medicine, Department of Medicine and Surgery, Azienda Ospedaliero-Universitaria di Parma, Via Gramsci 14, 43100 Parma, Italy; 4 “Paolo Ottonello” Psychiatry Institute, Via Pepoli 5, 40123 Bologna, BO Italy

**Keywords:** Negative symptoms, Clinical high risk, Early psychosis, Early intervention, Follow-up, Outcome

## Abstract

Negative symptoms in CHR-P people are generally not responsive to treatments and commonly related to poorer functional outcome. However, less research attention has been dedicated to Persistent Negative Symptoms (PNS), defined as clinically stable negative symptoms of moderate severity evident for at least 6 months. This study aims to (a) determine the prevalence of PNS in a sample of young people at CHR-P; (b) investigate any association of PNS with functioning and clinical features; (c) examine longitudinal course of PNS across 2 years of follow-up and changes in PNS severity levels with specialized treatments. One Hundred Eighty CHR-P participants were recruited and were divided into CHR-P/PNS + and CHR-P/PNS− subgroups. The clinical assessments were based on the PANSS and the GAF and were conducted at baseline and every 12 months during the follow-up. Twenty four participants showed PNS at entry. Of them, 21 concluded the 2-year follow-up period. At baseline, the CHR-P/PNS + participants showed more educational and employment deficits, and more social and functioning impairment. During the follow-up, the CHR-P/PNS + subgroup had a significant longitudinal decrease in negative symptoms, which was specifically related to antidepressant treatment. CHR-P/PNS + subjects also showed a higher incidence of new hospitalization and a lower functional recovery over time. Our findings support that the persistence of negative symptoms in CHR-P people is longitudinally related to worse daily functioning and more severe clinical conditions that are at higher risk of hospitalization and are less responsive to specialized treatments.

**Supplementary Information:**

The online version contains supplementary material available at 10.1007/s00406-024-01808-w.

## Introduction

There has been an increasing clinical and research interest in the last few years about Negative Symptoms (NS) in people with First Episode of Psychosis (FEP) and at Clinical High Risk for Psychosis (CHR-P). In this respect, recent evidence in CHR-P populations showed that NS are among the main determinants for psychosis transition [1, 2], as they often appear before other clinical features, are generally not responsive to treatments [3, 4], and correlate with poorer functional outcome [5, 6]. Moreover, continuous, frequent monitoring of NS in people at CHR-P seems also to be justified by another crucial empirical finding, i.e., the initial 6 months of treatment after the enrollment in specialized EIP programs is a key window for improving NS as less improvement is likely afterward [7].

Despite this growing interest in NS in early psychosis, less attention has been dedicated to Persistent Negative Symptoms (PNS), which are usually considered particularly difficult to address and to treat [8]. According to Buchanan (2007) [9], PNS are defined as clinically stable NS of moderate severity evident for an extended period [10]. Specifically, these following criteria are currently required: (a) presence of at least moderate (i.e., a score of ≥ 4 on the PANSS scale) for at least 3 NS or at least moderately severe (i.e., a score of ≥ 5 on the PANSS) for at least 2 NS; (b) persistence of NS for at least 6 months and for an extended period of time prior to the study beginning (e.g., at least 4 weeks); and (c) absence of relevant levels of positive symptoms, depression, and extrapyramidal symptoms as defined thresholds on accepted and validated rating scales [11, 12].

In studies examining the impact of PNS in people with FEP, it was reported that the presence of PNS is specifically related to increased decline in daily functioning [13–16], poorer quality of life [16], greater cognitive deficits [15], and longer duration of the prodromal phase [17].

Although research on PNS in CHR-P is still limited, increasing evidence recently showed that CHR-P individuals with PNS have poorer premorbid social adaptation compared to those without PNS, together with the presence of deficits in verbal fluency, processing speed, and a greater prevalence of childhood maltreatment (in particular, neglect) [18]. Other interesting investigations also showed more severe impairment in social and role functioning during the follow-ups in the CHR subgroup with PNS in comparison with CHR-P participants without PNS [10, 19]. However, knowledge about PNS in CHR-P individuals and their treatment response remains to be filled [20].

Starting to this background, the aims of this study were to (a) determine the prevalence of PNS in a sample of young people at CHR-P recruited within an Italian “Early Intervention in Psychosis” (EIP) program; (b) investigate any relevant association of PNS with sociodemographic data, functioning, and other relevant clinical features both at baseline and across a 2-year follow-up period; and (c) examine longitudinal course of PNS across the follow-up and any significant relationship of changes in PNS severity levels with the specialized treatment components provided within the EIP program, as well as with specific outcome parameters (e.g., psychosis transition, CHR-P criteria persistence, suicidal thoughts and behavior, new hospitalization, service disengagement, functional and symptomatic remission). We specifically hypothesized that CHR-P participants show significant deficits in functioning and more severe psychopathology, in line with the previous studies, and that PNS are associated with poorer prognosis compared to CHR-P individuals without PNS.

## Methods

### Sample and setting

CHR-P participants were consecutively recruited within the “Parma At-Risk Mental States” (PARMS) program from January 2016 to December 2021. The PARMS program is a specialized EIP infrastructure diffusely implemented across all adult and adolescent mental health services in the Parma Department of Mental Health (Northern Italy) [21].

Inclusion criteria were as follows: (a) specialist mental healthcare request; (b) age 12–25 years, (c) to meet CHR-P criteria as defined by the “Comprehensive Assessment of At-Risk Mental States” (CAARMS) [22] at the baseline assessment (i.e., Genetic Vulnerability, “Attenuated Psychotic Symptoms” [APS], or “Brief Limited Intermittent Psychotic Symptoms” [BLIPS]).

Exclusion criteria were as follows: (a) previous affective or non-affective psychotic episodes; (b) past exposure to AP medication or current AP intake exceeding 4 weeks in the present episode; (c) known intellectual disability (I.Q. < 70); and (d) neurological or medical disorder with psychiatric manifestations. Previous use of AP drug was considered a proxy for a past psychotic episode, consistently with the original CAARMS criteria for psychosis threshold [23]. A current AP prescription of less than 4 weeks was required to minimize pharmacological interference with baseline psychopathological assessment [24].

All participants and parents (if minors) provided written informed consent for their participation in the study. This research obtained approval from the local ethics committee (AVEN Ethics Committee protocol n. 559/2020/OSS*/AUSLPR) and adhered to the principles outlined in the 1964 Declaration of Helsinki and its later amendments.

### Instruments

The psychopathological assessment of this research included the CAARMS, the “Health of the Nation Outcome Scale” (HoNOS) [25], the “Positive and Negative Syndrome Scale” (PANSS) [26], and the “Global Assessment of Functioning” (GAF) scale [27].

The CAARMS is a clinical interview designed to explore various aspects of attenuated psychopathology. Its “Positive Symptoms” subscale was used for defining both CHR-P and psychosis criteria. CAARMS interviews were conducted by trained PARMS team members using the approved Italian version (CAARMS-ITA) [28]. Specifically, PARMS team members were fully trained from the main author of the CAARMS-ITA, who was trained at “Orygen,” the National Centre of Youth Mental Health” in Melbourne (Australia). Regular CAARMS scoring workshops and supervision sessions were implemented to ensure good-to-excellent interrater reliability [29]. Every 12 months in the follow-up, CAARMS interview was re-administered to psychometrically identify psychosis transition and CHR-P criteria persistence.

The HoNOS was developed to evaluate mental health and social functioning of individuals with severe mental illness, including patients with early psychosis [30]. As originally indicated by Wing and colleagues (1999), subscale scores were derived by grouping items into four main domains: (a) “Behavioral Problems,” (b) “Impairment,” (c) “Psychiatric Symptoms,” and (d) “Social Problems” [31]. According to Kortrijk and co-workers [32], we considered a score of ≤ 2 on the HoNOS items 9, 10, and 11 (included in the HoNOS “Social Problems” domain) as index of functional remission.

The PANSS is a widely used clinical interview for assessing psychopathology in psychosis, including young people with early psychosis [19, 33, 34]. PANSS interviews were conducted by trained PARMS team members with a long-term clinical experience (more than 15 years) with patients with early psychosis. Moreover, regular PANSS scoring workshops were repeated across the follow-up to ensure good-to-excellent interrater reliability (Intraclass Correlation Coefficient [ICC] values of > 0.75 for all item and factor scores). As indicated in the meta-analytic model proposed by Shafer and Dazzi [35], after checking its goodness of fit in our CHR-P total sample [11], we considered five principal psychopathological domains: “Disorganization,” “Negative Symptoms,” “Positive Symptoms,” “Resistance/Excitement-Activity,” and “Affect” (“Depression-Anxiety”).

As for the negative dimension, we, however, decided to use the PANSS negative symptom factor recommended by the European Psychiatric Association (EPA) [12], because it has proven to be more coherent with the current conceptualization of negative symptoms [8]. It includes the following 5 PANSS items: N1 “Blunted affect,” N2 “Emotional withdrawal,” N3 “Poor rapport,” N4 “Passive/apathetic social withdrawal,” and N6 “Lack of spontaneity and flow of conversation.” Compared to the EPA configuration, the negative symptom model proposed by Shafer and Dazzi [35] also encompassed PANSS G7 “Motor retardation” and G16 “Active social avoidance” items, which showed to be not really negative symptoms since they were more related to extrapyramidal symptoms, depression, suspiciousness, or social anxiety [12]. Additionally, to support this choice, we conducted a confirmatory factor analysis exploring three competitive models of different PANSS negative symptom factor configurations reported in the current literature on early psychosis (see Supplementary Materials [Table S1] for details). Since the EPA model also showed to have the best fit values in our CHR-P population across the follow-up period, we considered it in the main statistical analysis of the current study. As index of symptomatic remission, we considered a score of ≤ 3 (corresponding to mild severity or less) on the 8 PANSS items indicated by the “Remission in Schizophrenia Working Group’s criteria” [36]. This index was used for defining clinical remission in CHR-P populations along with the absence of persistence of CHR-P criteria at follow-up assessments [37, 38].

In accordance with Buchanan [9], conservative clinical criteria for PNS were used: (a) presence of at least moderate (i.e., score of ≥ 4 on the PANSS) for at least 3 negative symptoms or at least moderately severe (i.e., score of ≥ 5 on the PANSS) for at least 2 negative symptoms; (b) persistence of negative symptoms for at least 6 months and for an extended time period of at least 4 weeks prior to the study beginning; and (c) absence of significant levels of positive symptoms, depression, and extrapyramidal symptoms as defined thresholds on accepted and validated rating scales (i.e., persistent scores of ≤ 3 in all items included in the PANSS “Positive Symptoms” domain and in PANSS “Depression” and “Guilt Feelings” items for at least 6 months in the same time period, together with the absence of extrapyramidal symptoms requiring anticholinergic treatment).

The GAF is a commonly used instrument for evaluating clinical status and socio-occupational functioning in individuals with severe mental disorders, including early psychosis [39]. According to Yang and colleagues [40], we considered a GAF score of > 60 at follow-ups as a current index of functional remission.

Finally, we completed a sociodemographic and clinical chart, including information on employment, “Duration of Untreated Illness” (DUI), new suicide attempt and self-harm behavior, current suicidal ideation, functional recovery, and service disengagement. Specifically, DUI was defined as the time interval between the first psychiatric manifestation and the first pharmacological/psychosocial intervention [41]. Suicide attempt was defined as potentially injurious, self-inflicted behavior without a fatal outcome for which there was (implicit or explicit) evidence of intent to die, while self-harm behavior was intended as acts of deliberate self-harm or intoxication with alcohol or drugs, but where there was no clear intention to die [42]. Service disengagement was defined as a complete lack of contact or untraceable for at least 3 months despite a need of treatment [43]. Current suicidal ideation was detected as a score of ≥ 2 on item 4 (“Suicidality”) of the Brief Psychiatric Rating Scale (BPRS), corresponding at least to occasional suicidal thinking without specific plans [44]. Lastly, as proposed by Silva and Restrepo (2019), functional recovery was simply defined as return to work or school [45].

All assessment tools were administered both at baseline (T0) and every 12 months during the 2-year follow-up period (i.e., at 1- and 2-year assessment time [T1 and T2]).

### Procedures

The initial DSM-5 diagnosis was formulated by a minimum of two trained PARMS team members using the “Structured Clinical Interview for DSM-5 mental disorders” (SCID-5) [46]. Based on CAARMS and PANSS interviews, CHR-P participants with PNS were categorized as CHR-P/PNS + subgroup. CHR-P subjects without PNS at entry were included in the CHR-P/PNS− subsample. Specifically, as for the identification of the criterion b of PNS, the “persistence of negative symptoms for at least 6 months” was assessed re-administering the PANSS after 12 months of follow-up. Moreover, information on “the persistence of negative symptoms for an extended time period of at least 4 weeks prior to the study beginning” was collected at baseline from patients’ clinical interviews and/or their medical reports.

Within 3–4 weeks from the PARMS enrollment, CHR-P individuals were assigned to a multi-professional team, including a clinical psychologist, an early rehabilitation case manager, and a psychiatrist. As recommended by current EIP official guidelines [47, 48], AP medication should be prescribed to CHR-P subjects who had (a) a rapid decline in daily functioning, (b) a sudden escalation of overt psychotic symptoms, (c) an immediate risk of suicide or severe violence, or (d) who failed to respond to specialized psychosocial interventions. As first-line pharmacological treatment, low-dose atypical AP drugs were used [49].

Individual psychotherapy was shaped on the model by van der Gaag and co-workers [50] for young people at CHR-P, offering at least 15 sessions per year (each lasting 60 min) [39]. Family psychoeducation was adapted on the model by McFarlane [51] for CHR-P individuals, providing at least 10–12 sessions to each family [52]. Finally, a dedicated case manager was also provided to each subject/family to coordinate all interventions planned, especially those aimed at promoting a recovery-oriented early rehabilitation through at least 24 sessions provided along the 2 years of follow-up (each lasting 60 min) [53].

Information regarding medication (type and dosage), intensity of psychosocial interventions, psychopathology, functioning, and outcome parameters (e.g., service disengagement, psychosis transition, new hospitalization, etc.) was collected both at baseline and each 12 months during the 2 years of follow-up.

Between-group comparisons on sociodemographic, clinical, and treatment measures at baseline were first investigated. Longitudinal changes in PNS severity levels along the 2-year follow-up period was also examined in the CHR-P/PNS + subsample, as well as any relevant association of PNS changes with psychopathological variables and treatment response over time. Lastly, intergroup comparisons on treatment response and specific outcome parameters were investigated.

### Statistical analysis

The data analysis was conducted using the Statistical Package for Social Science (SPSS), version 15.0 for Windows [54]. All tests were two-tailed, with a *p* value significance set at 0.05. In intergroup comparisons, categorical variables were examined using the Chi-square (X2) test, while continuous variables using the Mann–Whitney U test.

Spearman’s rank correlation coefficients were calculated to investigate any significant associations of PANSS “Negative Symptoms” factor scores with sociodemographic and clinical parameters both at baseline and along the 2-year follow-up period (T2) in the CHR-P/PNS + subsample. The Wilcoxon test for repeated measures was also carried out to assess the longitudinal stability of PANSS NS factor scores in the CHR-P/PNS + subgroup across the 2 years of our follow-up.

Additionally, multiple linear regression analyses with PANSS NS factor scores as dependent variables and the specialized PARMS intervention components as independent variables were performed in the CHR-P/PNS + subsample. In our longitudinal examinations, we specifically used the differences (deltas [Δ]) between T0 and T1 or T2 PANSS, GAF, and HoNOS scores as primary clinical parameters to examine overtime. Indeed, according to Ver Hoef [55], the delta scores better describe longitudinal changes and temporal dynamics of psychopathology and the clinical status compared to T0, T1, and T2 single scores.

Furthermore, a mixed-design ANOVA was also carried out to investigate between-group comparisons on longitudinal treatment response for NS. Finally, as for time-to-event outcome parameters (i.e., psychosis transition, new hospitalization, new suicide attempt, new self-harm behavior, and service disengagement), after having previously checked that the proportionality-of-hazards assumption was met, univariate models were fitted for each outcome variable across the 2 years of follow-up using Cox regression analysis [56]. For not time-to-event dependent parameters (such as CHR-P criteria persistence, current suicidal ideation, functional recovery, GAF or HoNOS functional remission, PANSS symptomatic remission, and persistent negative symptoms), binary logistic regression analyses with PNS subgroups as independent variables were performed [57].

## Results

One hundred and eighty CHR-P patients were recruited for this study. Of them, 140 (77.8%) met APS criteria and 30 (16.7%) met BLIPS criteria (Table 1). Twenty-four (13.3%) CHR-P participants showed PNS at entry, with different DSM-5 diagnoses and symptoms (see Supplementary Materials [Figure S1] for details).

**Table 1:** Baseline sociodemographic and clinical comparisons between the two CHR-P subgroups

Variable	CHR-P/PNS + (n = 24)	CHR-P/PNS− (n = 156)	X2/z	*p*
Gender (males)	16 (66.7%)	74 (47.4%)	3.077	.079
Ethnic group (white Caucasian)	20 (83.3%)	139 (89.1%)	0.672	.491
Migrant status	5 (20.8%)	23 (14.7%)	0.587	.543
Civil status (single)	22 (91.6%)	153 (98.1%)	1.941	.267
Living status (with parents)	22 (91.6%)	146 (93.6%)	2.439	.486
NEET	15 (62.5%)	42 (26.9%)	6.467	**.019**
Age (at entry)	20.43 ± 3.46	19.39 ± 3.83	− 1.081	.28
Education (in years)	10.96 ± 2.62	11.38 ± 2.42	− 0.886	.375
DUI (in weeks)	41.22 ± 27.50	48.60 ± 50.86	− 0.145	.884
Past hospitalization	6 (25.0%)	22 (14.1%)	1.88	.222
Past specialist contact	13 (54.2%)	70 (44.9%)	0.723	.395
Past attempted suicide	1 (4.2%)	18 (11.5%)	1.197	.476
Family history of psychosis	8 (33.3%)	51 (32.7%)	0.004	.95
Current substance abuse	4 (16.7%)	27 (17.3%)	0.006	.999
*CHR-P subgroups*				
APS	19 (79.2%)	121 (77.6%)	0.031	.86
BLIPS	5 (20.8%)	25 (16.0%)	0.346	.56
Genetic vulnerability	0 (0.0%)	10 (6.4%)	1.039	.144
*PANSS score*				
Positive symptoms	11.79 ± 5.14	12.60 ± 6.70	− 0.074	.941
Negative symptoms	20.92 ± 4.56	13.02 ± 5.63	− 4.178	**.0001**
Disorganization	20.33 ± 6.96	15.15 ± 4.92	− 2.634	**.04**
Affect	15.00 ± 5.01	15.43 ± 5.23	− 0.432	.666
Resistance/excitement-activity	9.42 ± 4.81	7.02 ± 2.93	− 1.591	.112
Total score	87.50 ± 15.49	68.82 ± 16.63	− 3.47	**.001**
GAF score	42.50 ± 10.26	49.69 ± 7.98	− 3.117	**.002**
*HoNOS score*				
Behavioral problems	2.58 ± 2.26	2.69 ± 2.06	− 0.49	.624
Impairment	2.17 ± 2.04	2.19 ± 1.90	− 0.107	915
Psychiatric symptoms	8.71 ± 3.20	8.75 ± 3.26	− 0.205	.837
Social problems	7.50 ± 2.84	5.99 ± 3.82	− 2.32	**.04**
Antipsychotic medication prescription	14 (58.3%)	78 (50.0%)	0.578	.447
Equivalent dose of chlorpromazine (mg/day)	169.20 ± 132.34	158.12 ± 112.99	− 0.067	.947
Antidepressant medication prescription	4 (16.7%)	39 (25.0%)	0.794	.373
Equivalent dose of fluoxetine (mg/day)	36.30 ± 18.50	30.31 ± 18.69	− 0.496	.643
Individual psychotherapy	14 (60.9%)	84 (55.3%)	0.255	.614
Family psychoeducation	7 (30.4%)	56 (36.8%)	0.356	.551
Case management	16 (69.6%)	84 (55.3%)	1.669	.196

Frequencies (and percentages), means ± standard deviation, Chi-squared test (*X*^2^), and Mann–Whitney *U* test (*z*) values are reported. Bonferroni’s corrected *p* values are reported. Statistically significant *p* values are in bold

*CHR-P* clinical high risk for psychosis; *PNS* persistent negative symptoms; *CHR-P/PNS* + CHR-P individuals with PNS; *CHR-P/PNS−* CHR-P individuals without PNS; *NEET* not in education, employment, or training; *DUI* duration of untreated illness; *APS* attenuated psychotic symptoms; *BLIPS* brief limited intermittent psychotic symptoms; *PANSS* positive and negative syndrome scale; *GAF* global assessment of functioning; *HoNOS* health of the nation outcome scale; *p* statistical significance

### Baseline data

As for sociodemographic features, CHR-P/PNS + individuals had a higher prevalence rate of NEET (i.e., participants “Not [engaged] in Education, Employment, or Training”) in comparison with the CHR-P/PNS− group (Table 1).

Compared to CHR-P/PNS−, CHR-P/PNS + subjects showed higher PANSS “Negative Symptoms” and “Disorganization” dimension subscores, as well as higher PANSS total score. They also had a lower GAF score and a higher HoNOS “Social Problems” domain subscore. In the CHR-P/PNS + subgroup at entry (T0), no statistically significant correlations between severity levels in negative symptoms and other clinical and sociodemographic characteristics were found (Table 2).

**Table 2:** Baseline associations between PNS and sociodemographic/clinical data at baseline in the CHR-P/PNS + subgroup (n = 24)

Variable	PANSS “negative symptoms” factor score (*ρ*/z)	*p*
Gender	− .193	.209
Ethnic group (white Caucasian)	− .994	.306
Migrant status	− .999	.304
Age (at entry)	− .05	.878
Education (in years)	− .509	.091
DUI (in weeks)	− .212	.508
Past specialist contact	− 1.471	.141
Family history of psychosis	− .854	.461
Current substance abuse	− 1.022	.373
*PANSS score*		
Positive symptoms	.083	.799
Disorganization	.458	.134
Affect	− .194	.546
Resistance/excitement-activity	.17	.598
GAF score	− .259	.417
*HoNOS score*		
Behavioral problems	− .059	.855
Impairment	.028	.931
Psychiatric symptoms	− .046	.888
Social problems	− .117	.717

Spearman rank correlation (*ρ*) and Mann–Whitney U test (*z*) values are reported. Bonferroni’s corrected *p* values are reported

*CHR-P* clinical high risk for psychosis; *PNS* persistent negative symptoms; *CHR-P/PNS* + CHR-P participants with PNS; *PANSS* positive and negative syndrome scale; *DUI* duration of untreated illness; *PANSS* positive and negative syndrome scale; *GAF* global assessment of functioning; *HoNOS* health of the nation outcome scale; *p* statistical significance

### Longitudinal data in the CHR-P/PNS + group

All 24 CHR-P/PNS + participants reached the 1-year assessment time (T1), while only 21 concluded the 2-year follow-up period (T2) (see Supplementary Materials [Figure S2] for details).

During the 2 years of follow-up, CHR-P/PNS + individuals showed a significant decrease in negative symptom severity levels. As no relevant longitudinal change in negative symptom severity was found during the second year of intervention but exclusively in the first 12 months, this reduction seemed to be primarily attributable to the first year of treatment (i.e., from T0 to T1) (Table 3).

**Table 3:** Longitudinal course of PANSS “Negative Symptoms” factor scores in the CHR-P/PNS + subgroup across the 2-year follow-up period

Variable	T0 score (n = 24)	T1 score (n = 23)	T2 score (n = 21)	T0–T2 delta score (*p*)	T0–T1 delta score (*p*)	T1–T2 delta score (*p*)
PANSS “Negative Symptoms” factor scores	20.92 (4.56)	18.33 (4.16)	17.50 (3.92)	− 2.023 (**.049**)	2.501 (**.012**)	− .654 (.513)

Mean (standard deviation), Wilcoxon test (*z*), Spearman rank correlation (*ρ*), and Mann–Whitney U test (*z*) values are reported. Bonferroni’s corrected *p* values are reported. Statistically significant *p* values are in bold

*CHR-P* clinical high risk for psychosis; *PNS* persistent negative symptoms; *CHR-P/PNS* + CHR-P participants with PNS; *PANSS* positive and negative syndrome scale; *T0* baseline assessment time; *T1* 1-year assessment time; *T2* 2-year assessment time; *p* statistical significance

Moreover, CHR-P/PNS + participants had significant positive correlations of the difference (delta) in T0–T2 PANSS “Negative Symptoms” factor subscores with deltas in T0–T2 PANSS “Disorganization” factor subscores and T1 equivalent dose of fluoxetine, as well as a relevant negative correlation with level of education (in years) (Table 4).

**Table 4:** Longitudinal association between PANSS “Negative Symptoms” factor scores and other clinical parameters in the CHR-P/PNS + subgroup across the 2-year follow-up period

Variable (n = 21)	T0–T2 PANSS “negative symptoms” factor score (*ρ*/z)	*p*
Gender	− .085	.117
Ethnic group (white Caucasian)	− .925	.355
Migrant status	− .923	.366
Age (at entry)	− .025	.946
Education (in years)	− .697	**.025**
DUI (in weeks)	− .382	.276
Past specialist contact	− .172	.422
Family history of psychosis	− .965	.352
Current substance abuse	− .394	.711
*T0–T2 PANSS scores*		
Positive symptoms	.608	.072
Disorganization	.812	**.020**
Affect	.45	192
Resistance/excitement-activity	− .033	.993
T0–T2 GAF score	− .305	.392
*T0–T2 HoNOS score*		
Behavioral problems	.242	.5
Impairment	.488	.153
Psychiatric symptoms	.502	.14
Social problems	.56	.092
T0 Equivalent dose of chlorpromazine (mg/day)	.203	.059
T1 Equivalent dose of chlorpromazine (mg/day)	.042	.678
T2 Equivalent dose of chlorpromazine (mg/day)	.005	.962
T0 Equivalent dose of fluoxetine (mg/day)	.108	.285
T1 Equivalent dose of fluoxetine (mg/day)	.229	**.049**
T2 Equivalent dose of fluoxetine (mg/day)	.176	.08
T2 number of individual psychotherapy sessions	.203	.062
T2 number of family psychoeducation sessions	.026	.794
T2 number of case management sessions	− .032	.751

Mean (standard deviation), Wilcoxon test (*z*), Spearman rank correlation (*ρ*), and Mann–Whitney U test (*z*) values are reported. Bonferroni’s corrected *p* values are reported. Statistically significant *p* values are in bold

*CHR-P* clinical high risk for psychosis; *PNS* persistent negative symptoms; *CHR-P/PNS* + CHR-P participants with PNS; *PANSS* positive and negative syndrome scale; *DUI* duration of untreated illness; *GAF* global assessment of functioning; *HoNOS* health of the nation outcome scale; *T0* baseline assessment time; *T1* 1-year assessment time; *T2* 2-year assessment time; *p* statistical significance

In the CHR-P/PNS + group, our linear regression analysis results exclusively showed that the delta in T0–T1 PANSS “Negative Symptoms” factor subscores was significantly predicted by T1 equivalent dose of fluoxetine (Table 5). No other predictive factor for longitudinal changes in negative symptom severity levels was observed.

**Table 5:** PANSS “Negative Symptoms” factor scores and their associations with the specialized treatment components of the PARMS program in the CHR-P/PNS + subgroup across the 2-year follow-up period

T0–T1 PANSS “Negative Symptoms” factor score (n = 23)	B	SE	β	p	95% CI		R2 = .978 F [df = 7] = 114.321 *p* = **.021**
					Lower	Upper	
Constant	.769	.91	–	.464	− 2.118	3.643	
T0 equivalent dose of chlorpromazine (mg/day)	1.812	.682	.606	.089	− .450	3.871	
T1 equivalent dose of chlorpromazine (mg/day)	.38	.352	.162	.399	− .819	1.567	
T0 equivalent dose of fluoxetine (mg/day)	− .003	.007	− .131	.381	− .022	.019	
T1 equivalent dose of fluoxetine (mg/day)	.045	.006	.653	**.019**	.017	.072	
T1 number of individual psychotherapy sessions	− .092	.118	− .221	.479	− .431	.259	
T1 number of family psychoeducation sessions	.354	.299	.263	.369	− .620	1.249	
T1 number of case management sessions	.031	.013	.107	.418	− .034	.071	

T1–T2 PANSS “negative symptoms” factor score (n = 21)	B	SE	β	p	95% CI		R2 = .680 F [df = 7] = .607 *p* = .741
					Lower	Upper	
Constant	3.755	6.691	–	.631	− 25.034	32.545	
T1 equivalent dose of chlorpromazine (mg/day)	.246	1.265	.179	.864	− 5.198	5.689	
T2 equivalent dose of chlorpromazine (mg/day)	− .885	1.566	− .465	.629	− 7.622	5.852	
T1 equivalent dose of fluoxetine (mg/day)	− .004	.023	− .122	.872	− .104	.095	
T2 equivalent dose of fluoxetine (mg/day)	− .006	.022	− .211	.8	− .101	.089	
T2 number of individual psychotherapy sessions	.126	.309	.705	.722	− 1.202	1.455	
T2 number of family psychoeducation sessions	− .3	.555	− .908	.643	− 2.689	2.089	

T0–T2 PANSS “negative symptoms” factor score (n = 21)	B	SE	β	p	95% CI		R2 = .758 F [df = 9] = .392 *p* = .851
					Lower	Upper	
Constant	2.008	12.59	–	.899	− 157.961	161.977	
T0 equivalent dose of chlorpromazine (mg/day)	.842	.876	.564	.233	.123	1.982	
T1 equivalent dose of chlorpromazine (mg/day)	.915	2.391	.436	.767	− 29.468	31.298	
T2 equivalent dose of chlorpromazine (mg/day)	− .949	3.018	− .326	.806	− 39.298	37.401	
T0 equivalent dose of fluoxetine (mg/day)	− .047	.083	− .748	.673	− 1.098	1.005	
T1 equivalent dose of fluoxetine (mg/day)	.007	.045	.137	.899	− .568	.583	
T2 equivalent dose of fluoxetine (mg/day)	.024	.064	.508	.776	− .794	.841	

Statistically significant *p* values are in bold.

*PANSS* positive and negative syndrome scale; *PARMS* parma at-risk mental states; *CHR-P* clinical high risk for psychosis; *PNS* persistent negative symptoms; *CHR-P/PNS* + CHR-P participants with PNS; *T0* baseline assessment time; *T1* 1-year assessment time; *T2* 2-year assessment time; *B* regression coefficient; *SE* standard error; *95% CI* 95% confident intervals for B, *β* standardized regression coefficient; *p* statistical significance; *R2* R-squared or coefficient of determination; *F* statistic test value for linear regression analysis; *df* degrees of freedom

Our mixed-design ANOVA results confirmed a significant time effect in the CHR-P total sample (“within-subject” effect) with a relevant longitudinal decrease in PANSS “Negative Symptoms” factor subscore (Table 6). However, we found a statistically significant group effect (“between-group” effect) in favor CHR-P/PNS− individuals. Specifically, the detailed examination of estimated marginal means showed significantly higher scores in negative symptom within the CHR/PNS + subgroup compared to the CHR/PNS + one along the entire follow-up (see Supplementary Materials [Figure S3] for profile plots of the two CHR-P subgroups).

**Table 6:** Mixed-design ANOVA results: PANSS “Negative Symptoms” factor scores across the 2-year follow-up period in the two CHR-P subgroups

Variable	Time effect				Group effect				Interaction effect (time x group)			
	d*f*	*F*	*p*	*η*^2^	d*f*	*F*	*p*	*η*^2^	d*f*	*F*	*p*	*η*^2^
PANSS “negative symptoms” factor scores	1.5	14.391	**.0001**	.128	1	34.041	**.0001**	.258	1.5	.108	.845	.001

Variables	CHR-P/PNS + (n = 21)				CHR-P/PNS− (n = 132)			
	T0 EMM (SE)	T1 EMM (SE)	T2 EMM (SE)	*t* (*p*)	T0 EMM (SE)	T1 EMM (SE)	T2 EMM (SE)	*t* (*p*)
PANSS “negative symptoms” factor scores	21.40 (1.78)	18.70 (1.67)	17.50 (1.37)	2.092 (.066)	13.67 (.593)	9.93 (.556)	9.13 (.457)	6.929 (**.0001**)

As all Mauchly’s tests of sphericity are statistically significant (*p* < 0.05), Greenhouse–Geisser corrected degrees of freedom to assess the significance of the corresponding *F* value are used. Statistically significant *p* values are in bold. Statistical trends in *p* value (*p* < 0.01) are underlined

*ANOVA* analysis of variance; *CHR-P* clinical high risk for psychosis; *PNS* persistent negative symptoms; *CHR-P/PNS* + CHR-P participants with PNS; *CHR-P/PNS−* CHR-P participants without PNS; *PANSS* positive and negative syndrome scale; *df* degrees of freedom; *F* F statistic value; *GAF* global assessment of functioning; *HoNOS* health of the nation outcome scale; *p* statistical significance; *η*^*2*^ partial eta squared; *T0* baseline assessment; *T2* 2-year assessment time; *EMM* estimated marginal mean; *SE* Standard error; *t* Student’s *t* statistical value

As for outcome parameters, at both T1 and T2 assessment, CHR-P/PNS + participants showed higher rates of new hospitalization compared to the CHR-P/PNS− subgroup (Table 7), as well as lower rates of GAF and HoNOS functional remissions (Table 8). Moreover, exclusively at T1, they also showed lower incidence rates of current suicidal ideation and functional recovery.

**Table 7:** Univariate Cox proportional-hazard models for 2-year time-to-event outcome parameters in the two CHR-P subgroups

Variable	CHR-P/PNS + (n = 24)	CHR-P/PNS− (n = 156)	Statistic test			
			HR	95% CI		*p*
				Lower	Higher	
1-year psychosis transition	1 (4.2%)	21 (13.5%)	0.04	0.001	10.043	0.254
1-year new hospitalization	9 (37.5%)	15 (9.6%)	3.965	1.735	9.061	**0.001**
1-year new suicide attempt	2 (8.3%)	6 (3.8%)	2.023	0.445	10.914	0.333
1-year new self-harm	4 (16.7%)	24 (15.4%)	1.101	0.382	3.174	0.858
1-year service disengagement	2 (8.3%)	9 (5.8%)	1.465	0.317	6.782	0.625
2-year psychosis transition	2 (8.3%)	27 (17.3%)	0.462	0.11	1.944	0.292
2-year new hospitalization	9 (37.5%)	17 (10.9%)	3.598	1.603	8.079	**0.002**
2-year suicide attempt	2 (8.3%)	11 (7.1%)	1.194	0.265	5.378	0.818
2-year self-harm behavior	4 (16.7%)	16 (10.3%)	0.648	0.28	1.502	0.312
2-year service disengagement	3 (12.5%)	24 (15.4%)	0.825	0.249	2.741	0.754

Suicide attempt = potentially injurious, self-inflicted behavior without a fatal outcome for which there was (implicit or explicit) evidence of intent to die, derived from direct information reported by the patient (or by a relative well informed about the facts) or documented in the clinical notes; Self-harm behavior = acts of deliberate self-harm or intoxication with alcohol or drugs, but where there was no clear intention to die. Service disengagement = complete lack of contact or untraceable for at least 3 months despite a need of treatment, counted from the date of the last face-to-face meeting with the clinical staff. Significant statistical *p* values are in bold

*CHR-P* clinical high risk; *PNS* persistent negative symptoms; *CHR-P/PNS* + CHR-P individuals with PNS; *CHR-P/PNS−* CHR-P individuals without PNS; *HR* hazard ratio; *95% CI* 95% confidence intervals for HR; *p* statistical significance

**Table 8:** Binary logistic regression analysis results for 2-year not time-to-event outcome variables by CHR-P subgroup

Dependent variable	CHR-P/PNS + (n = 24)	CHR-P/PNS− (n = 156)	Statistic test				
			B (SE)	HR	95% CI		p
					Lower	Higher	
1-year CHR-P criteria persistence	14 (58.3%)	69 (44.5%)	− .577 (.444)	.573	.24	1.369	.21
1-year current suicidal ideation	3 (13.6%)	61 (4.7%)	1.468 (.643)	4.341	1.231	15.311	**.022**
1-year functional recovery	6 (25.0%)	97 (62.2%)	1.596 (.499)	4.932	1.853	13.128	**.001**
1-year GAF functional remission	1 (4.2%)	105 (67.3%)	3.858 (.1.036)	47.353	6.22	36.504	**.0001**
1-year HoNOS functional remission	5 (2.8%)	123 (78.8%)	2.651 (.540)	14.164	4.92	4.776	**.0001**
1-year PANSS symptomatic remission	12 (52.2%)	85 (55.1%)	.568 (.444)	1.765	.739	4.217	.201
2-year CHR-P criteria persistence	12 (5.0%)	49 (31.4%)	− .781 (.443)	.458	.192	1.092	.078
1-year current suicidal ideation	6 (25.0%)	59 (37.8%)	.601 (.499)	1.825	.686	4.857	.229
2-year functional recovery	13 (54.2%)	103 (66.0%)	.497 (.443)	1.644	.69	3.29	.262
2-year GAF functional remission	7 (29.2%)	109 (69.9%)	1.729 (.482)	5.632	2.191	14.481	**.0001**
2-year HoNOS functional remission	14 (58.3%)	133 (85.3%)	1.418 (.472)	4.13	1.639	1.41	**.003**
2-year PANSS symptomatic remission	15 (62.5%)	120 (76.9%)	.693 (.462)	2	.808	4.951	.134

Current suicidal ideation = score of ≥ 2 on item 4 (“Suicidality”) of the Brief Psychiatric Rating Scale (BPRS), corresponding at least to occasional suicidal thinking without specific plans; Functional recovery = return to work/school; GAF functional remission = GAF score ≥ 60; HoNOS functional remission = HoNOS item 9, 10 and 11 subscores > 2; PANSS symptomatic remission = PANSS item P1, P2, P3, N1, N4, N6, G5, G9 subscores ≤ 3. Significant statistical* p* values are in bold

*CHR-P* clinical high risk; *PNS* persistent negative symptoms; *CHR-P/PNS* + CHR-P individuals with PNS; *CHR-P/PNS−* CHR-P individuals without PNS; *GAF* global assessment of functioning; *HoNOS* health of the nation outcome scale; *PANSS* positive and negative syndrome scale; *B* regression coefficient; *SE* standard error; *HR* hazard ratio; *95% CI* 95% confidence intervals for HR; *p* statistical significance

## Discussion

The results of this investigation showed that the baseline prevalence of PNS in the PARMS program was about 13%. This finding is slightly higher than what was reported in similar CHR-P samples, ranging from 6 to 9% [10, 18]. The difference in PNS prevalence may be related to different criteria for defining clinical persistence of negative features and/or different assessment instruments for measuring negative symptoms across studies. Specifically, the “North American Prodrome Longitudinal Study-2” (NAPLS-2) applied a PNS definition exclusively based on having one of the three main negative symptoms of the “Scale of Psychosis-risk Symptoms” (i.e., social anhedonia, avolition, and expression of emotion) scored ≥ 4 (i.e., moderately severe to extreme) for a duration of 1 year [19]. Differently, at the PACE (“Personal Assessment and Clinical Evaluation”) Clinic in Melbourne (Australia) [18], the authors defined PNS as presence of at least one global “Negative symptoms” subscale score (measured with the “Scale for the Assessment of Negative Symptoms” [SANS]) of ≥ 3 at baseline and at follow-up, a combined total score of 6 or less on the BPRS (“Brief Psychiatric Rating Scale”) subscales of depression, guilt, and suicidality (corresponding to an average of “very mild” or less on each item), and a combined total score of 16 or less on the BPRS psychotic subscales of conceptual disorganization, hallucinations, suspiciousness, and unusual thought content (corresponding to an average of “moderate” or less on each item), without using any measure of extrapyramidal symptoms.

As for assessment instruments for measuring negative symptoms, it is important to specify that the PANSS is not strictly in line with the current conceptualization of negative symptoms [12]. Specifically, it includes few negative characteristics and only focuses on the patient’s behavior, failing to evaluate the subject’s internal experience. Moreover, this instrument was developed to be mainly used for adult individuals with full-blown psychotic features, and is probably not enough sensitive to detect the subtle manifestations of negative symptoms in CHR-P individuals. In this respect, second generation scales (such as the “Brief Negative Symptom Scale” [BNSS]) [58] are more in line with current conceptualizations of negative symptoms and have versions more adapted to the intended population. Therefore, future research using second generation scale to investigate PNS is needed.

However, given the high diffusion of the PANSS in clinical populations with early psychosis, our results have the potential to be replicated in similar populations, and this is of great clinical relevance since investigations examining beneficial effects of specialized interventions on PNS are still relative poor. Furthermore, it is important to consider that assessment of PANSS G6 “Depression” and G3 “Guilt feelings” items is not completely adequate to exclude the presence of significant depressive symptoms, as the absence of anticholinergic treatment is not enough to exclude the presence of Parkinsonism. In this respect, both the original definition of PNS [9] and the EPA [12] recommended the use of accepted and validated rating scales, such as the “Calgary Depression Scale” or the “Hamilton Depression Rating Scale” to assess depressive features, and the “Simpson-Angus Extrapyramidal Rating Scale” or the “Extrapyramidal Symptom Rating Scale” to evaluate extrapyramidal symptoms.

In line with what was observed by Yung et al. [18] in the NAPLS-2 population, our CHR-P/PNS + participants showed more educational and employment deficit at baseline compared to the CHR-P/PNS− group, as well as more social and functioning impairment. These findings confirm evidence on PNS in patients with first episode psychosis, presenting statistically significant associations with greater unemployment rates and poorer executive functioning at entry [13, 59]. Moreover, this impairment in daily functioning seemed to be stable over time. Indeed, compared to CHR-P/PNS−, CHR-P/PNS + participants showed lower GAF scores and higher HoNOS “Social Problems” domain subscores also at both T1 and T2 assessment (see Supplementary Materials [Table S2] for details). In this sense, PNS remain a relevant, enduring “stigma” for a satisfying functional and social recovery in this young population [11].

In comparison with CHR-P/PNS−, CHR-P/PNS + individuals also had more severe levels of psychopathology at entry, especially in terms of higher PANSS scores in negative symptoms and disorganization. Additionally, this group-specific greater clinical severity appeared to be persistent over time as confirmed at both T1 and T2 assessment (see Supplementary Materials [Table S2] for details). Overall, these findings confirm that PNS may be considered as enduring psychopathological indicators of clinical severity and poorer clinical recovery.

Across the 2 years of our follow-up, the CHR-P/PNS + subgroup showed a relevant longitudinal decrease in negative symptom severity levels (especially during the first year of intervention). This was specifically correlated with lower educational levels, higher equivalent dose of fluoxetine at T1, and a longitudinal improvement in disorganization (as well as in T0–T2 delta PANSS total scores calculated without negative symptom item subscores [*ρ* = 0.600; *p* = 0.009]). These findings confirm a general poor school performance and a privileged psychopathological relationship between negative symptoms and disorganization, in line with the Bleuler’s thinking on “basic” symptoms in schizophrenia [60]. This special link could thus be established before full-blown psychotic symptoms emerge and already characterize the prodromal phase of psychosis, perhaps as direct, early features of a psychotic vulnerability [61].

The results of our multiple linear regression analysis also showed that the longitudinal decrease in PNS severity (specifically in the first year of treatment) was significantly predicted by T1 antidepressant dosage. No association with antipsychotic medication or other psychosocial interventions was found. Moreover, when considering the two-factor structure of negative symptoms described in the literature (i.e., “Expressive” and “Experiential” subdomains) using the PANSS model proposed by Jang and co-workers [62] (i.e., “Expressive” factor including PANSS N1, N3, N6, and G7 items, and “Experiential” factor including PANSS N2, N4, and G16 items), our regression analysis results showed that only longitudinal severity decrease in “Expressive” subdimension (especially during the first year of intervention) was significantly predicted by T1 antidepressant dosage (*B* = 0.012; *p* = 0.017), together with a higher session number of family psychoeducation (*B* = 0.558; *p* = 0.043) (see Supplementary Materials [Table S3] for details).

Unfortunately, the beneficial effect related to the use of antidepressant drug did not extent into the second year of treatment, and seems to be particularly helpful to improve the diminished expression domain (specifically including blunted affect and alogia) [63], together with the provision of family psychoeducation. This finding seems to be an isolated result in the current literature, especially when independent from improvement in depressive features. Indeed, there is only evidence that a combined therapy with antidepressant in addiction to antipsychotic medication showed beneficial effects on negative symptoms in people with schizophrenia [64, 65]. In this respect, recent research has confirmed that different antidepressants among “Selective Serotonin Re-uptake Inhibitors” (SSRIs) could have different effects [66]. Therefore, we hope to conduct clinical research with more participants in the near future, looking forward to analysis and clinical application of the differences in these pharmacological effects. As for how antidepressant dosage at T1 assessment can predict a negative symptom change between T0 and T1, it is probably related to the pharmacological treatment maintenance across the 1-year follow-up period. Indeed, all CHR-P/PNS + participants who were prescribed with antidepressant drug at baseline, continued to take this medication steadily after one year of intervention (see Supplementary Materials [Table S2] for details).

However, as there were multiple regression models conducted for the three time periods but fluoxetine dosage was only significant in T0–T1 period, this effect seems to be poorly robust and requires further confirmation in both larger CHR-P/PNS + populations and longer prospective research. Moreover, although we excluded clinically relevant severity levels of PANSS “Depression” and “Guilt feelings” scores from the PNS definition, the beneficial effect of fluoxetine in the first year of treatment may also affect other potential secondary negative symptoms (such as blunted affect and avolition). Therefore, future studies using more specific assessment instrument for clinical depression in psychosis are needed. Anyway, our result on fluoxetine effects remarks the importance of an early recognition and a timely intervention focused on expressed negative symptoms.

In this respect, few investigations were prospectively designed to evaluate the effect of pharmacological treatment on PNS, with most reports of decrease in negative symptoms being based on short-term research [67, 68]. Meta-analytic results on randomized, controlled antipsychotic drug trials in patients with schizophrenia reported that low-dose amisulpride (approved for negative symptoms in some European countries) was the only antipsychotic that was superior to placebo in the treatment of predominant negative symptoms. However, a parallel improvement of depression was also observed, making it difficult to evaluate whether the decrease in negative symptoms was due to reduction in clinical depression. Furthermore, a more recent prospective (26 weeks), randomized controlled trial showed that significant differences and clinically relevant improvement in negative symptoms were demonstrated in favor of cariprazine over risperidone [69]. In this study, changes in symptoms from other domains were small, indicating that improvement was specific to negative symptoms and not related to changes in positive, extrapyramidal, and depressive symptoms. Given the considerable unmet medical need associated with negative symptoms [70], drug development is active in this therapeutic area for agents with activity at different receptor systems including NMDA receptors, alpha 7 nicotinic receptors, and 5-HT2A and sigma-2 receptors [71].

Given the limited effective pharmacologic treatments to treat negative symptoms, mental health professionals should be aware of psychosocial interventions that can be used together with antipsychotic medication. In line with our finding on the beneficial effect of family psychoeducation on the expressive subdomain of negative symptoms, some authors suggested that interventions aimed at addressing attitudes, behaviors, and poor psychosocial functioning (including a focus on healthy lifestyles, with emphasis on exercise, sleep, diet, smoking cessation, appropriate alcohol consumption, and social participation) may help individuals gain insight into how their symptoms affect their outlook [72]. In this respect, while social skills training and cognitive remediation therapy showed some evidence for negative symptom improvement [73, 74], the most widely studied psychological intervention is Cognitive-Behavioral Therapy (CBT). Specifically, CBT demonstrated a positive (but moderate) effect on negative symptoms, with a reduction of apathy and improved motivation [75], as well as it seems to support awareness of the link between a patient’s thoughts, behaviors, and feelings in an effort to change symptoms and functioning [4]. Finally, family interventions may help patients and family members cope with the burden of negative symptoms through psychoeducation, communication training, behavioral problem solving, and crisis management [76]. Despite mixed and inconsistent findings, referring patients to psychosocial intervention could be an important way for mental health professionals to support patients and their families as they cope with negative symptoms and attempt to improve outcomes and quality of life [33]. However, future, prospective clinical trials are needed to improve the evidence base for psychosocial interventions in negative symptoms of psychosis (especially in PNS).

Comparing the two CHR/P subgroups, our mixed-design ANOVA results showed a statistically relevant “between-group effect” within global evidence of a significant “time effect” (“within-subjects effect”) in PANSS “Negative Symptoms” score decrease over time (especially in the first year of intervention) (see Supplementary Materials [Table S4] for details). As hypothesized, CHR/PNS− participants showed lower negative symptom severity levels across the entire follow-up period in comparison with the CHR-P/PNS + subgroup, suggesting that the persistence of negative symptoms in CHR-P individuals may negatively interfere not only with the severity of clinical course over time but also with treatment response on negative dimension. In this respect, the longitudinal improvement in negative symptom severity levels found in our CHR-P/PNS− subgroup could also be due to higher beneficial effects of PARMS interventions on decrease in secondary negative symptoms (especially those related to positive symptoms, suspiciousness, and clinical depression).

As for outcome parameters, CHR-P/PNS + subjects showed higher incidence rates of new hospitalization, lower functional remission, and lower functional recovery compared to CHR-P/PNS− ones. All these findings specifically support that the persistence of negative symptoms in people at CHR-P is longitudinally related to worse daily functioning, poorer real-world-performance, and more severe clinical conditions that are at higher risk of hospital admission and are less responsive to the specialized treatments usually provided within EIP services.

### Strengths and limitations

This investigation notably examined PNS (defined using conservative clinical criteria) in a clinical sample of young individuals at CHR-P, (i.e., without evident full-blown psychotic features) treated within an EIP service across a 2-year follow-up period. This gave us the opportunity to strictly monitor the progression of negative symptoms over time and to verify their clinical persistence and their treatment response.

However, some limitations should also be considered. A first weakness of our research was related to the small sample size (especially for the CHR-P/PNS + subgroup). This did not allow a reliable generalization of our results and requires replication in future perspective studies on larger CHR-P populations.

Second, although conservative clinical criteria were used [9], the original definition of PNS would require the use of accepted and validated rating scales to evaluate depression and extrapyramidal symptoms (such as the “Calgary Depression Scale” or the “Hamilton Depression Rating Scale,” and the “Simpson-Angus Extrapyramidal Rating Scale” or the “Extrapyramidal Symptom Rating Scale”). Therefore, our assessment of PANSS “Depression” and “Guilt feelings” items may not have been enough to exclude the presence of depressive symptoms, as the absence of anticholinergic treatment could be insufficient to exclude the presence of Parkinsonism. Future research using more specific assessment instruments for extrapyramidal and depressive symptoms is thus needed.

Third, as there was a general reduction in intensity of psychosocial intervention sessions offered within the PARMS program during the second year of treatment [77], this may have contributed to worsening longitudinal outcome, independently to the greater, intrinsic treatment-resistant nature of PNS and their related poorer care response [78]. Thus, maintaining an adequate intensity of EIP treatments also during the second/third year of intervention could consolidate the longitudinal decrease of PNS and more successfully promote functional recovery of this young population. However, in order to more strongly support that the reported longitudinal improvement in PNS severity levels is due to the effect of our specialized treatments, future research with control comparisons is needed. In this respect, other comparable CHR observation studies without interventions found that similar symptom improvements could be seen within the first 6–12 months [79].

Fourth, we examined CHR-P subjects in a “real-world” clinical setting primarily involved in the delivery of evidence-based care pathways within standard community mental healthcare centers. Therefore, our findings should be compared only with similar clinical populations.

Moreover, the current investigation was conducted within a specialized CHR-P program not specifically focused on the evaluation of negative symptoms. In particular, negative symptoms were assessed with the PANSS, a psychometric scale commonly used also in CHR-P populations [2, 11, 80, 81], but poorly articulated and composed of only seven negative symptom items. Therefore, future research using more specific, second generation instruments for negative symptoms (such as the “Brief Negative Symptom Scale” [BNSS] or the “Clinical Assessment Interview for Negative Symptoms” [CAINS]) [58, 82] that were developed to rectify shortcomings of older scales, is needed. Moreover, it is well known that the PANSS was not developed to address negative symptoms in CHR individuals. In this respect, specific scales (such as the “Negative Symptom Inventory” [NSI]) [83] have been developed specifically for the CHR-P population and should be used in future investigations. However, given the high diffusion of the PANSS in populations with early psychosis, our results have the potential to be replicated in similar populations, and this is of great clinical relevance since investigations examining beneficial effects of specialized interventions on PNS are still relative poor.

Additionally, although widely used in CHR-P individuals, also the GAF conflates symptoms and functioning, and cannot be considered an optimal measure of daily functioning, especially in terms of real-world socio-occupational performance. Therefore, future research using more specific instruments to assess social and occupational functioning (such as the “Global functioning social and role scales”) [84] is needed. As for providing more valid statistical results on the association between these potentially overlapping constructs, we also conducted further correlation analyses with the GAF using the EPA negative symptom factor model [85]. However, no statistically significant correlations between baseline GAF score and baseline PANSS “Negative Symptoms” factor subscore suggested by the EPA was observed (*ρ* = − .179; *p* = .599), as well as between T0–T2 delta in GAF scores and T0–T2 delta in PANSS “Negative Symptoms” factor subscores (*ρ* = − .312; *p* = .380).

Finally, in comparing our results with both existing and future empirical evidence, it is necessary to take into account the PNS criteria used to dichotomize CHR-P individuals in the present research. Indeed, different PNS criteria may create CHR-P subgroups with poorly comparable psychopathological characteristics.

## Conclusions

PNS are clinically significant also in a minority of young people at CHR-P treated within specialized EIP programs. Although the results of this investigation show a relevant association between PNS and poorer functional outcomes in young people at CHR-P during a 2-year follow-up period, a longitudinal decrease over time was also observed. Specifically, this reduction seems to be related to specific EIP treatments (such as the T1 antidepressant dosage). In this respect, future research should aim to improve the early identification of CHR people and focus on PNS in a larger portion of population at risk, with a special attention to intensive treatments and rehabilitation programs during the follow-up period.

### Supplementary Information

Below is the link to the electronic supplementary material.

Supplementary file1 (DOCX 86 KB)

## Data Availability

The data that support the findings of this investigation are available from the corresponding author upon reasonable request. The data are not publicly available due to privacy/ethical restrictions.
