# Supplementary material for: Persistent negative symptoms in young people at clinical high risk of psychosis treated with an Italian early intervention program: a longitudinal study
Source: Eur Arch Psychiatry Clin Neurosci. 2024 Apr 26;274(6):1311–26. doi: 10.1007/s00406-024-01808-w (PMC11362215; doi:10.1007/s00406-024-01808-w)
Supplement: Supplementary file 1 — Supplementary file1 (DOCX 86 KB) [file 406_2024_1808_MOESM1_ESM.docx]

Table S1 – CFA indices of adjustment in the CHR-P total sample (n = 180): exploring competitive models of different PANSS negative symptom factor configurations.

| NSF factor model | CFI | TLI | RMSEA | SRMR | AIC |
| --- | --- | --- | --- | --- | --- |
| T0  7-item NSF  5-item NSF  8-item NSF  T1  7-item NSF  5-item NSF  8-item NSF  T2  7-item NSF  5-item NSF  8-item NSF | .957  **.975**  .921  .990  **.999**  .962  .991  **.999**  .899 | .936  **.950**  .890  .985  **.999**  .946  .986  **.999**  .901 | .111  .175  .174  .097  **.010**  .149  .090  **.034**  .187 | .082  **.065**  .112  .058  **.020**  .098  .067  **.032**  .123 | 2703.894  **2039.934**  2992.733  2442.129  **1763.112**  2531.388  1893.460  **1347.411**  1931.388 |

Note - CFA = Confirmatory Factor Analysis; PANSS = Positive And Negative Syndrome Scale; CHR-P = Clinical High Risk for Psychosis; NSF = Negative Symptom Factor; χ^2^ = Chi-squared test value; df = degrees of freedom; p = statistical significance; T0 = baseline assessment; CFI = Comparative Fit Index; TLI = Tucker-Lewis Index; RMSEA = Root Mean Square Error of Approximation; SRMR = Standardized Root Mean Square Residual; AIC = Akaike Information Criterion; 7-item NSF = NSF model including PANSS N1, N2, N3, N4, N6, G7, and G16 items (Shafer and Dazzi, 2019); 5-item NSF = NSF model including PANSS N1, N2, N3, N4, and N6 items (Galderisi et al., 2021); 8-item NSF = NSF including PANSS N1, N2, N3, N4, N6, N7, G5, and G10 items specifically identified in a CHR-P population in Singapore (Yang et al., 2018). Statistically best fit values are in bold.

In **CFA**, we applied the robust weighted least squares estimator that does not assume normally distributed parameters and provides the best option for modeling ordinal data in moderately large samples. The criterion of Brown (2006) was used to explain CFA results. It recommends four common indices to assess ft of the overall model and to calculate the model adjustment: Comparative Fit Index (CFI), Tucker‐Lewis Index (TLI), Root Mean Square Error of Approximation (RMSEA) and Standardized Root Mean Square Residual (SRMR). According to Hu and Bentler (1999), the following general rules of thumb were considered in this study: TLI/CFI > 0.90 (accepted fit), RMSEA <0.08 (accepted fit) and SRMR<0.08 (good fit). Additionally, we calculated the Akaike Information Criterion (AIC), a parsimony correction index to find the most parsimonious model which fitted the data. Smaller AIC scores were indicative of the preferred model in terms of comparison to fit and parsimony (Brown, 2006).

**References**:

Shafer A, Dazzi F (2019) Meta-analysis of the Positive and Negative Syndrome Scale (PANSS) factor structure. J Psychiatr Res 115: 113-120.

Galderisi S, Mucci A, Dollfus S, Nordentoft M, Falkai P, Kaiser S, Giordano GM, Vandevelde A, Nielsen MO, Glenthøj LB, Sabé M, Pezzella P, Bitter I, Gaebel W (2021) EPA guidance on assessment of negative symptoms in schizophrenia. Eur Psychiatry 64: e23.

Yang Z, Lim K, Lam M, Keefe R, Lee J (2018) Factor structure of the positive and negative syndrome scale (PANSS) in people at ultra-high risk (UHR) for psychosis. Schizophr Res 201: 85-90.

Brown TA (2006) Confirmatory factor analysis for applied research. Guilford Press, New York, NY

Hu L, Bentler PM (1999) Cutof criteria for ft indexes in covari#ance structure analysis: conventional criteria versus new alterna#tives. Struct Equ Modeling 6:1–55.

Figure S1 – Persistent negative symptoms in the CHR-P group at baseline.

180 CHR-P participants

24 (13.3%) with PNS

156 (86.7%) without PNS

8 (33.3%) Depressive disorder

5 (20.8%) Schizotypal personality disorder

4 (16.7%) Brief psychotic disorder

3 (12.5%) Anxiety disorder

2 (8.3%) Psychotic disorder NOS

1 (4.2%) Borderline personality disorder

1 (4.2%) OCD

Note – CHR-P = Clinical High Risk for Psychosis; PNS = Persistent Negative Symptoms; NOS = Not Otherwise Specified; OCD = Obsessive-compulsive disorder.

20 (30.3%) Depressive disorder

10 (15.2%) Psychotic disorder NOS

8 (12.2%) OCD

8 (12.1%) Brief psychotic disorder

7 (10.6%) Anxiety disorder

6 (9.1%) borderline personality disorder

5 (7.6%) schizotypal personality disorder

2 (3.0%) Eating disorder

20 (30.3%) Depressive disorder

10 (15.2%) Psychotic disorder NOS

8 (12.2%) OCD

8 (12.1%) Brief psychotic disorder

7 (10.6%) Anxiety disorder

6 (9.1%) borderline personality disorder

5 (7.6%) schizotypal personality disorder

2 (3.0%) Eating disorder

Figure S2 – Service disengagement across the 2-year follow-up period in the CHR-P total group (n = 180).

24 CHR-P/PNS+

156 CHR-P/PNS-

180 CHR-P participants

175 CHR-P participants

5 service disengagement

23 CHR-P/PNS+

152 CHR-P/PNS-

22 service disengagement

153 CHR-P participants

132 CHR-P/PNS-

21 CHR-P/PNS+

Note – CHR-P = Clinical High Risk for Psychosis; PNS = Persistent Negative Symptoms; CHR-P/PNS+ = CHR-P individuals with PNS; CHR-P/PNS- = CHR-P/PNS- = CHR-P individuals without PNS; T0 = baseline assessment time; T1 = 1-year assessment time; T2 = 2-year assessment time.

20 (30.3%) Depressive disorder

10 (15.2%) Psychotic disorder NOS

8 (12.2%) OCD

8 (12.1%) Brief psychotic disorder

7 (10.6%) Anxiety disorder

6 (9.1%) borderline personality disorder

5 (7.6%) schizotypal personality disorder

2 (3.0%) Eating disorder

20 (30.3%) Depressive disorder

10 (15.2%) Psychotic disorder NOS

8 (12.2%) OCD

8 (12.1%) Brief psychotic disorder

7 (10.6%) Anxiety disorder

6 (9.1%) borderline personality disorder

5 (7.6%) schizotypal personality disorder

2 (3.0%) Eating disorder

Figure S3 – Mixed-design ANOVA profile plots for PANSS “Negative Symptoms” factor scores across the 2-year follow-up period in the two CHR-P subgroups.


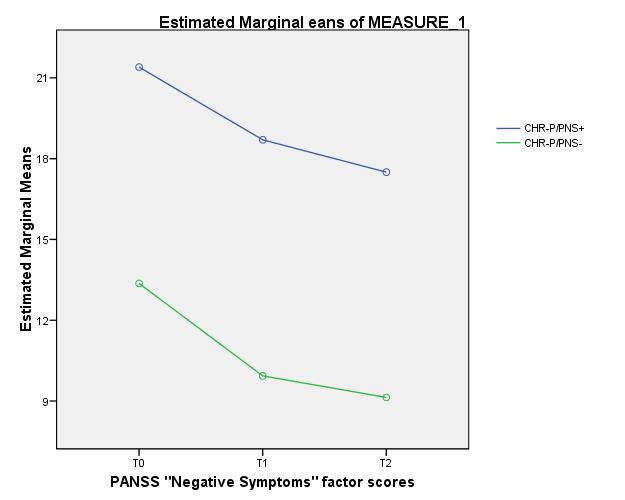


Note. ANOVA = Analysis of Variance; PANSS = Positive And Negative Syndrome Scale; CHR-P = Clinical High Risk for Psychosis; PNS = Persistent Negative Symptoms; CHR-P/PNS+ = CHR-P participants with PNS; CHR-P/PNS- = CHR-P participants without PNS; T0 = baseline assessment time; T1 = 1-year assessment time; T2 = 2-year assessment time.

Table S2 – Sociodemographic and clinical comparisons between the two CHR-P subgroups across the follow-up.

| Variable at T1 | CHR-P/PNS+  (n = 23) | CHR-P/PNS-  (n = 152) | X^2^/z | p |
| --- | --- | --- | --- | --- |
| Gender (males)  Ethnic group (white Caucasian)  Migrant Status  Age (at entry)  Education (in years)  *PANSS score*  Positive symptoms  Negative symptoms  Disorganization  Affect  Resistance/excitement-activity  Total score  GAF score  *HoNOS score*  Behavioral problems  Impairment  Psychiatric symptoms  Social problems  Antipsychotic medication prescription  Equivalent dose of chlorpromazine (mg/day)  Antidepressant medication prescription  Equivalent dose of fluoxetine (mg/day)  Individual psychotherapy  Family psychoeducation  Case management | 16 (69.6%)  19 (82.6%)  4 (17.4%)  20.13±3.14  10.87±2.60  10.13±2.13  18.33±4.16  17.58±4.89  13.42±4.94  9.92±5.21  78.75±15.06  44.29±10.07  2.05±2.20  2.14±2.20  6.57±3.32  7.29±2.88  11 (47.8%)  60.00±36.00  6 (26.1%)  32.66±30.42  14 (60.9%)  7 (30.4%)  16 (69.6%) | 70 (46.1%)  136 (89.5%)  20 (13.2%)  19.29±3.85  11.40±2.42  9.68±4.25  9.85±5.26  14.06±5.70  12.02±4.76  6.84±2.99  58.59±20.76  61.71±12.33  1.46±1.60  1.36±1.50  5.59±3.38  3.86±2.93  73 (48.0%)  58.87±35.77  33 (21.7%)  31.14±33.23  84 (55.3%)  56 (36.8%)  84 (55.3%) | 4.419  .930  .303  -.977  -1.117  -981  -4.671  -2.496  -.945  -1.754  3.440  -4.541  -1.014  -1.505  -1.500  -4.456  .001  -.294  .221  -.476  .255  .356  1.669 | **.036**  .306  .527  .328  .264  .327  **.0001**  **.043**  .345  .395  **.001**  **.0001**  .311  .132  .134  **.0001**  .986  .769  .638  .634  .614  .551  .196 |
| Variable at T2 | CHR-P/PNS+  (n = 21) | CHR-P/PNS-  (n = 132) | X^2^/z | p |
| Gender (males)  Ethnic group (white Caucasian)  Migrant Status  Age (at entry)  Education (in years)  *PANSS score*  Positive symptoms  Negative symptoms  Disorganization  Affect  Resistance/excitement-activity  Total score  GAF score  *HoNOS score*  Behavioral problems  Impairment  Psychiatric symptoms  Social problems  Antipsychotic medication prescription  Equivalent dose of chlorpromazine (mg/day)  Antidepressant medication prescription  Equivalent dose of fluoxetine (mg/day)  Individual psychotherapy  Family psychoeducation  Case management | 14 (66.7%)  17 (81.0%)  4 (19.0%)  20.24±3.81  10.95±2.71  9.00±3.81  17.50±3.92  16.20±3.46  13.40±5.02  9.30±4.08  74.20±13.09  48.58±11.18  1.52±1.75  1.19±1.12  5.52±3.20  5.62±2.97  11 (52.4%)  8 (38.1%)  14 (66.7%)  9 (42.9%)  16 (76.2%) | 63 (47.7%)  120 (90.9%)  16 (12.2%)  19.51±3.91  11.52±2.49  9.68±4.17  9.11±4.35  13.78±5.29  11.80±4.55  6.57±2.87  56.91±20.28  64.15±12.45  1.21±1.40  1.16±1.33  4.83±3.07  3.57±2.81  62 (47.0%)  30 (22.7%)  78 (59.1%)  56 (42.4%)  82 (62.1%) | 2.600  1.918  .765  -.721  -1.098  -.086  -4.366  .2.042  -.986  .1.810  -3.042  -3.758  -.558  -.456  -.981  -2.997  .213  2.292  .434  .001  1.558 | .107  .239  .382  .471  .272  .932  **.0001**  **.049**  .324  .142  **.002**  **.0001**  .557  .648  .326  **.012**  .645  .130  .510  .970  .212 |

Note. CHR-P = Clinical High Risk for Psychosis; PNS = Persistent Negative Symptoms; CHR-P/PNS+ = CHR-P individuals with PNS; CHR-P/PNS- = CHR-P individuals without PNS; T1 = 1-year assessment time; T2 = 2-year assessment time; PANSS = Positive And Negative Syndrome Scale; GAF = Global Assessment of Functioning; HoNOS = Health of the Nation Outcome Scale; p = statistical significance. Frequencies (and percentages), means ± standard deviation, Chi-squared test (X^2^) and Mann-Whitney U test (z) values are reported. Bonferroni’s corrected p values are reported. Statistically significant p values are in bold.

Table S3 - PANSS “Negative Symptoms” subdomain scores and their associations with the specialized treatment components of the PARMS program in the CHR-P/PNS+ subgroup across the 2-year follow-up period.

| T0-T1 PANSS NSF “Expressive” subdomain  (n = 24) | B | SE | | | β | | | p | | 95% CI  Lower Upper | | R^2^ = .978  F _[df = 7]_ = 19.300  p =**.017** |
| --- | --- | --- | --- | --- | --- | --- | --- | --- | --- | --- | --- | --- |
| Constant  T0 equivalent dose of chlorpromazine (mg/day)  T1 equivalent dose of chlorpromazine (mg/day)  T0 equivalent dose of fluoxetine (mg/day)  T1 equivalent dose of fluoxetine (mg/day)  T1 number of individual psychotherapy sessions  T1 number of family psychoeducation sessions  T1 number of case management sessions | .596  1.860  -.189  -.002  .012  -.169  .558  -.006 | .511  .384  .212  .003  .005  .061  .165  .009 | | | -  1.119  -.136  -.093  .319  -.737  .809  -.075 | | | .328  .069  .438  .473  **.017**  .069  **.043**  .511 | | -1.032  .640  -.863  -.012  -.002  -.363  .031  -.034 | 2.224  3.081  .485  .007  .027  .024  1.084  .021 |  |
| T1-T2 PANSS NSF “Expressive” subdomain  (n = 21) | B | | SE | β | | | p | | 95% CI  Lower Upper | | | R^2^ = .904  F _[df = 7]_ = 1.283  p =.505 |
| Constant  T1 equivalent dose of chlorpromazine (mg/day)  T2 equivalent dose of chlorpromazine (mg/day)  T1 equivalent dose of fluoxetine (mg/day)  T2 equivalent dose of fluoxetine (mg/day)  T2 number of individual psychotherapy sessions  T2 number of family psychoeducation sessions  T2 number of case management sessions | -.746  .424  -.579  -.039  .044  .026  -.031  -.003 | | 1.379  .595  .518  .061  .064  .048  .094  .005 | -  .497  -.572  -1.673  1.901  .357  -.205  -.224 | | | .643  .550  .380  .588  .565  .643  .775  .577 | | -6.680  -2.136  -2.807  -.303  -.233  -.180  -.434  -.023 | | 5.188  2.983  1.649  .225  .322  .232  .373  .017 |  |
| T0-T2 PANSS NSF “Expressive” subdomain  (n = 21) | B | | SE | | | β | p | | 95% CI  Lower Upper | | | R^2^ = .987  F _[df = 9]_ = 8.435  p = .065 |
| Constant  T0 equivalent dose of chlorpromazine (mg/day)  T1 equivalent dose of chlorpromazine (mg/day)  T2 equivalent dose of chlorpromazine (mg/day)  T0 equivalent dose of fluoxetine (mg/day)  T1 equivalent dose of fluoxetine (mg/day)  T2 equivalent dose of fluoxetine (mg/day)  T2 number of individual psychotherapy sessions  T2 number of family psychoeducation sessions  T2 number of case management sessions | 2.997  1.425  -.043  .100  -.016  -.111  .014  -.077  .103  -.013 | | .932  .606  .532  .499  .004  .032  .006  .029  .067  .003 | | | -  .630  .523  .045  -.444  .074  .276  -.484  .315  -.423 | .085  .143  .299  .860  .069  .521  .132  .117  .262  .057 | | -1.015  -1.181  -.099  -2.046  -.034  -.234  -.010  -.202  -.184  -.025 | | 7.008  4.030  .299  2.246  .003  .198  .039  .048  .390  .001 |  |

| T0-T1 PANSS NSF “Experiential” subdomain  (n = 24) | B | SE | | | β | | | p | | 95% CI  Lower Upper | | R^2^ = .880  F _[df = 7]_ = 3.146  p =.188 |
| --- | --- | --- | --- | --- | --- | --- | --- | --- | --- | --- | --- | --- |
| Constant  T0 equivalent dose of chlorpromazine (mg/day)  T1 equivalent dose of chlorpromazine (mg/day)  T0 equivalent dose of fluoxetine (mg/day)  T1 equivalent dose of fluoxetine (mg/day)  T1 number of individual psychotherapy sessions  T1 number of family psychoeducation sessions  T1 number of case management sessions | .166  -.151  .562  -.003  .030  .081  -.246  .021 | 1.064  .798  .440  .006  .009  .127  .344  .018 | | | -  -.103  .456  -.129  .879  .398  -.404  .276 | | | .886  .862  .292  .660  .052  .568  .525  .327 | | -3.220  -2.691  -.840  -.023  .000  -.322  -1.341  -.037 | 3.552  2.388  1.963  .017  .060  .483  .848  .079 |  |
| T1-T2 PANSS NSF “Experiential” subdomain  (n = 21) | B | | SE | β | | | p | | 95% CI  Lower Upper | | | R^2^ = .941  F _[df = 7]_ = 4.574  p =.191 |
| Constant  T1 equivalent dose of chlorpromazine (mg/day)  T2 equivalent dose of chlorpromazine (mg/day)  T1 equivalent dose of fluoxetine (mg/day)  T2 equivalent dose of fluoxetine (mg/day)  T2 number of individual psychotherapy sessions  T2 number of family psychoeducation sessions  T2 number of case management sessions | -1.412  .088  -1.070  -.055  .078  .136  -.173  -.004 | | 1.069  .461  .402  .048  .050  .037  .073  .004 | -  .076  -.774  -1.730  2.453  1.374  -.849  -.211 | | | .318  .866  .117  .365  .261  .067  .141  .388 | | -6.013  -1.897  -2.797  -.260  -.138  -.024  -.485  -.019 | | 3.190  2.073  .658  .150  .293  .296  .140  .011 |  |
| T0-T2 PANSS NSF “Experiential” subdomain  (n = 21) | B | | SE | | | β | p | | 95% CI  Lower Upper | | | R^2^ = .995  F _[df = 9]_ = 25.378  p = .152 |
| Constant  T0 equivalent dose of chlorpromazine (mg/day)  T1 equivalent dose of chlorpromazine (mg/day)  T2 equivalent dose of chlorpromazine (mg/day)  T0 equivalent dose of fluoxetine (mg/day)  T1 equivalent dose of fluoxetine (mg/day)  T2 equivalent dose of fluoxetine (mg/day)  T2 number of individual psychotherapy sessions  T2 number of family psychoeducation sessions  T2 number of case management sessions | -.437  -.620  -.566  -.693  -.008  -.067  .093  .149  -.226  -.006 | | .504  .408  .344  .273  .002  .014  .015  .018  .038  .002 | | | -  -.443  -.499  -.501  -.354  -2.110  2.924  1.510  -1.113  -.327 | .545  .370  .287  .239  .191  .127  .105  .078  .106  .162 | | -6.838  -5.803  -5.333  -4.158  -.038  -.241  -.104  -.085  -.711  -.026 | | 5.965  4.362  3.221  2.773  .023  .106  .289  .383  .258  .014 |  |

Note – PANSS = Positive And Negative Syndrome Scale; NSF = Negative Symptom Factor; PARMS = Parma At-Risk Mental States; CHR-P = Clinical high Risk for Psychosis; PNS = Persistent Negative Symptoms; CHR-P/PNS+ = CHR-P participants with PNS. T0 = baseline assessment time; T1 = 1-year assessment time; T2 = 2-year assessment time; B = regression coefficient, SE = Standard Error, 95% CI = 95% Confident Intervals for B, β = standardized regression coefficient; p = statistical significance, R^2^ = R-squared or coefficient of determination, F = statistic test value for linear regression analysis, df = degrees of freedom. Statistically significant p values are in bold.

According to Jang and co-workers (2016), The NSF “Expressive” subdomain exclusively included PANSS N1 “Blunted affect”, N3 “Poor rapport”, N6 “Lack of spontaneity/flow of conversation”, and G7 “Motor retardation” items, while the NSF “Experiential exclusively included PANSS N2 “Emotional withdrawal”, N4 “Passive social withdrawal”, and G16 “Active social avoidance” items.

Reference:

Jang SK, Choi HI, Park S, Jaekal E, Lee GY, Cho YI, Choi KH (2026) A Two-Factor Model Better Explains Heterogeneity in Negative Symptoms: Evidence from the Positive and Negative Syndrome Scale. Front Psychol 7: 707. https://doi.org/10.3389/fpsyg.2016.00707.

Table S4 – Mixed-design ANOVA results: PANSS “Negative Symptoms” factor scores across the 2 follow-up periods (T0-T1 and T1-T2) in the CHR-P total groups.

| Variable | Time effect  (between T0 and T1) | | | | Time effect  (between T1 and T2) | | | |
| --- | --- | --- | --- | --- | --- | --- | --- | --- |
|  | df | F | p | η^2^ | df | F | p | η^2^ |
| PANSS “Negative Symptoms” factor scores | 1 | 16.959 | **.0001** | .124 | 1 | 2.965 | .088 | .029 |

Note. ANOVA = analysis of variance; CHR-P = Clinical High Risk for Psychosis; PANSS = Positive And Negative Syndrome Scale; df = degrees of freedom; F = F statistic value; GAF = Global Assessment of Functioning; HoNOS = Health of the Nation Outcome Scale; p = statistical significance; η^2^ = partial eta squared; T0 = Baseline assessment; T2 = 2-year assessment time.

As all Mauchly’s tests of sphericity are statistically significant (p<0.05), Greenhouse–Geisser corrected degrees of freedom to assess the significance of the corresponding F value are used. Statistically significant p values are in bold. Statistical trends in p value (p<0.01) are underlined.
